# Supplementary material for: MetaRibo-Seq measures translation in microbiomes
Source: Nat Commun. 2020 Jun 29;11:3268. doi: 10.1038/s41467-020-17081-z (PMC7324362; doi:10.1038/s41467-020-17081-z)
Supplement: Supplementary file 10 — Supplementary Data 7 [file 41467_2020_17081_MOESM10_ESM.zip › File2/Confidence_High_Taxonomy/222969_out.krona.html]

Javascript must be enabled to view this page.

members
magnitude
magnitudeUnassigned
count
unassigned
taxon
rank

222969\_out

18

18
superkingdom
2

phylum
18
1239

class
18
186801

order
18
186802

18
family
186806

18
genus
1730


SRS016267\_contig\_number\_1548
species
1
142586

39485
species
17

SRS013951\_contig\_number\_26210SRS017247\_contig\_number\_contig-100\_262.34113SRS017307\_contig\_number\_contig-100\_673.177009SRS019693\_contig\_number\_1957SRS024663\_contig\_number\_8782SRS042628\_contig\_number\_14103SRS063985\_contig\_number\_29492SRS142923\_contig\_number\_17143SRS143085\_contig\_number\_4430SRS143417\_contig\_number\_7826SRS144537\_contig\_number\_20462SRS146764\_contig\_number\_29506SRS147919\_contig\_number\_17538SRS148159\_contig\_number\_58044SRS148970\_contig\_number\_3604SRS149181\_contig\_number\_30279SRS893295\_contig\_number\_7169
